# Effects of beta-blockers on quality of life and well-being in patients with myocardial infarction and preserved left ventricular function—a prespecified substudy from REDUCE-AMI

**DOI:** 10.1093/ehjcvp/pvae062

**Published:** 2024-09-01

**Authors:** Katarina Mars, Sophia Humphries, Philip Leissner, Martin Jonsson, Patric Karlström, Jörg Lauermann, Joakim Alfredsson, Thomas Kellerth, Annica Ravn-Fischer, David Erlinge, Bertil Lindahl, Troels Yndigegn, Tomas Jernberg, Claes Held, Erik M G Olsson, Robin Hofmann

**Affiliations:** Department of Clinical Science and Education, Division of Cardiology, Karolinska Institutet, Södersjukhuset, Stockholm, Sjukhusbacken 10, 188 83, Sweden; Department of Women's and Children's Health, Uppsala University, Uppsala 751 85, Sweden; Department of Women's and Children's Health, Uppsala University, Uppsala 751 85, Sweden; Department of Clinical Science and Education, Division of Cardiology, Karolinska Institutet, Södersjukhuset, Stockholm, Sjukhusbacken 10, 188 83, Sweden; Department of Internal Medicine, Ryhov County Hospital, Jönköping 551 85 Sweden; Department of Internal Medicine, Ryhov County Hospital, Jönköping 551 85 Sweden; Department of Cardiology and Department of Health, Medicine and Caring Sciences, Linköping University, Linköping 581 83, Sweden; Division of Cardiology and Emergency medicine, Centralsjukhuset Karlstad, Karlstad 651 82, Sweden; Department of Cardiology, Sahlgrenska University Hospital, Gothenburg 405 30, Sweden; Institute of Medicine, Department of Molecular and Clinical Medicine, Sahlgrenska Academy University of Gothenburg, Gothenburg 405 30, Sweden; Department of Cardiology, Clinical Sciences, Lund University, Skane University Hospital, Lund 222 42, Sweden; Department of Medical Sciences, Cardiology, Uppsala University, Uppsala 751 85, Sweden; Uppsala Clinical Research Center, Uppsala University, Uppsala 751 85, Sweden; Department of Cardiology, Clinical Sciences, Lund University, Skane University Hospital, Lund 222 42, Sweden; Department of Clinical Sciences, Danderyd Hospital, Karolinska Institutet, Stockholm 171 77, Sweden; Department of Medical Sciences, Cardiology, Uppsala University, Uppsala 751 85, Sweden; Uppsala Clinical Research Center, Uppsala University, Uppsala 751 85, Sweden; Department of Women's and Children's Health, Uppsala University, Uppsala 751 85, Sweden; Department of Clinical Science and Education, Division of Cardiology, Karolinska Institutet, Södersjukhuset, Stockholm, Sjukhusbacken 10, 188 83, Sweden

**Keywords:** AMI: Acute myocardial infarction, EQ-VAS: EuroQoL visual analogue scale, EQ-5D: EuroQoL 5-dimension, LVEF: Left ventricular ejection fraction, QoL: Quality of life, SWEDEHEART: Swedish Web System for Enhancement and Development of Evidence-based care in Heart disease Evaluated According to Recommended Therapies, WHO-5: World Health Organization-5

## Abstract

**Aims:**

In the Randomized Evaluation of Decreased Usage of Beta-Blockers after Acute Myocardial Infarction (REDUCE-AMI) study, long-term beta-blocker use in patients after acute myocardial infarction (AMI) with preserved left ventricular ejection fraction demonstrated no effect on death or cardiovascular outcomes. The aim of this prespecified substudy was to investigate effects of beta-blockers on self-reported quality of life and well-being.

**Methods and results:**

From this parallel-group, open-label, registry-based randomized clinical trial, EQ-5D, and World Health Organization well-being index-5 (WHO-5) questionnaires were obtained at 6–10 weeks and 11–13 months after AMI in 4080 and 806 patients, respectively. We report results from intention-to-treat and on-treatment analyses for the overall population and relevant subgroups using Wilcoxon rank sum test and adjusted ordinal regression analyses. Of the 4080 individuals reporting EQ-5D (median age 64 years, 22% female), 2023 were randomized to beta-blockers. The main outcome, median EQ-5D index score, was 0.94 [interquartile range (IQR) 0.88, 0.97] in the beta-blocker group, and 0.94 (IQR 0.88, 0.97) in the no-beta-blocker group 6–10 weeks after AMI, OR 1.00 [95% CI 0.89–1.13; *P* > 0.9]. After 11–13 months, results remained unchanged. Findings were robust in on-treatment analyses and across relevant subgroups. Secondary outcomes, EQ-VAS and WHO-5 index score, confirmed these results.

**Conclusion:**

Among patients after AMI with preserved left ventricular ejection fraction, self-reported quality of life and well-being was not significantly different in individuals randomized to routine long-term beta-blocker therapy as compared to individuals with no beta-blocker use. These results appear consistent regardless of adherence to randomized treatment and across subgroups which emphasizes the need for a careful individual risk-benefit evaluation prior to initiation of beta-blocker treatment.

## Graphical Abstract

**Graphical Abstract:**
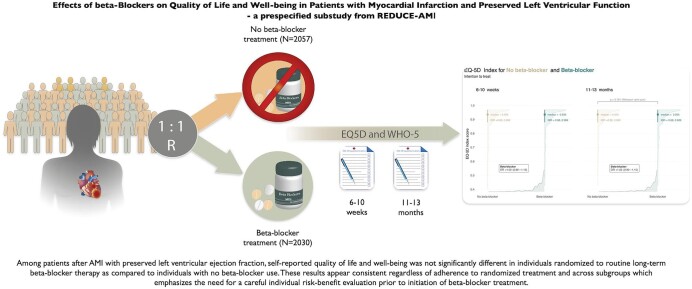

## Introduction

For decades, beta-blockers have been an essential part of routine secondary preventive treatment for all patients surviving acute myocardial infarction (AMI).^1^ The landmark trials showing that long-term beta-blocker therapy reduces mortality by approximately 20%^2–5^ were, however, performed before the introduction of early reperfusion treatment with revascularization and antithrombotic agents when patients suffered large myocardial infarcts commonly leading to the development of heart failure. In a meta-analysis incorporating trials from the reperfusion era, beta-blockers did not significantly reduce mortality.^6^ Beta-blockers are also known to cause clinically relevant adverse effects of hypotension, bradycardia, and more subjective symptoms like fatigue, exercise intolerance, and deterioration of psychosocial functioning, which potentially counterbalance clinical benefits and worsen quality of life (QoL) and well-being.^7–10^ Observational data on the possible influence of beta-blockers on patients’ overall QoL have yielded conflicting results, and data from randomized clinical trials (RCTs) are lacking.^11,12^ Noteworthy, reduced QoL after AMI has been associated with more frequent re-hospitalizations, and has been identified as a predictor of subsequent cardiovascular events and all-cause death.^13^

The recent Randomized Evaluation of Decreased Usage of Beta-Blockers after Acute Myocardial Infarction (REDUCE-AMI) trial showed that long-term use of beta‑blockers in patients with AMI and preserved left ventricular ejection fraction (LVEF ≥50%) did neither reduce the risk of subsequent cardiovascular endpoints including death compared with no beta‑blocker use nor increase hospitalizations for adverse events.^14^ How these results impact on clinical practice and guidelines remains to be seen at this point.^15^ However, experts are debating if routine initiation of beta-blockers in similar AMI patients should be abolished, and if beta-blocker treatment should be discontinued in individuals already treated with these agents.^16^ In this context, data on the QoL and well-being may be crucial for optimal future recommendations.

To enable a comprehensive risk-benefit assessment, this substudy investigated the effect of initiating long-term beta-blocker treatment on self-reported QoL and well-being during short- and long-term follow-up. We present results from the intention-to-treat (ITT) and on-treatment analyses for the overall population and across relevant subgroups.

## Method

### Study design

We performed a prespecified nationwide multicentre substudy of the REDUCE-AMI trial, a prospective, open-label, parallel-group, and registry-based RCT carried out in three countries: Sweden (38 centres), Estonia (1 centre), and New Zealand (6 centres). The design and rationale of the main trial and its primary results have been published previously.^14,17^ The current substudy was conducted at all 38 sites in Sweden with routine follow-up in the Swedish Web-system for Enhancement and Development of Evidence-based care in Heart disease Evaluated According to Recommended Therapies (SWEDEHEART) registry.^18^ At 8 of these sites, we collected additional data on psychological distress, sexual dysfunction, and QoL.^19^ Here, we present data on self-reported levels of QoL and well-being.

The trial was approved by the ethical review authority in Sweden (Dnr: 2016/1707–31/4 and 2018/1048–32).

### Patient population

Inclusion in the REDUCE-AMI trial was mandatory before assessment for eligibility in this substudy. The REDUCE-AMI trial included adult patients after written informed consent up to 7 days after AMI who had a preserved LVEF (≥50%) on echocardiography, and coronary angiography showing obstructive coronary artery disease (i.e. stenosis of ≥50%, a fractional flow reserve of ≤0.80, or instantaneous wave-free ratio of ≤0.89 in any segment) before randomization. Major exclusion criteria were an indication for or contraindication to beta-blocker treatment.

On top of these criteria, participants from the 8 hospitals collecting additional QoL data were required to have good understanding of the Swedish language to be eligible.

### Study procedure and data collection

After providing written informed consent, patients were randomly assigned to either beta-blocker treatment or no beta-blocker treatment in a 1:1 fashion with permuted blocks using an internet-based randomization module. The responsible physician was encouraged to aim for at least 100 mg Metoprolol or 5 mg Bisoprolol for those randomized to beta-blocker treatment. Patients who were already on beta-blocker before accepting participation and randomized to no beta-blocker received a tapering-schedule over 2–4 weeks, depending on earlier dosage. Patients were encouraged to continue the use of beta-blockers after discharge until the eventual occurrence of a contraindication. Patients who were randomized to the no-beta-blocker group were discouraged from using beta-blockers as long as there was no other indication than secondary prevention after AMI.

The patient population for this substudy was derived from the SWEDEHEART registry,^18^ including data routinely collected for all AMI patients in Sweden, with the age limit for follow-up visits below 75 years until 2018 and below 80 years thereafter. SWEDEHEART encompasses data from the hospital stay (age, sex, time and site variables, diagnosis of AMI, past and present medications, hospital treatment variables, comorbidities, demographic data, and more) and at two routine follow-up visits (6–10 weeks and 11–13 months after AMI) including the EuroQol 5-dimension (EQ-5D) questionnaire^20^ and present medication (beta-blockers and other cardiovascular therapies) among other variables. Doses or reasons for treatment changes are not reported. SWEDEHEART has excellent nationwide coverage (100% of cardiac care units, >90% of patients with myocardial infarction (MI) <80 years). Data are monitored regularly with >95% agreement between the registry and health records.^18^

Well-being was evaluated in a subgroup of patients from 8 centres using the World Health Organization-5 well-being index questionnaire (WHO-5)^21^ (details about patient population and data collection is provided in Supplementary Appendix).^19^

Information on date of death or emigration was obtained from the Swedish population registry.^22^ Diagnoses of atrial fibrillation, heart failure, bradycardia, second or third-degree AV-block, syncope, or hospitalization due to asthma or chronic obstructive pulmonary disease were obtained from the national patient registry, a mandatory registry including all ICD codes for all admission to hospitals in Sweden.^23^ Linkage with the national registries and the study data base was performed at the end of follow up by the Swedish National board of health and welfare.

### Outcomes

The main outcome of this substudy was EQ-5D index score by randomization status. The EQ visual analog scale (EQ-VAS) and WHO-5 index score were secondary outcomes.

The EQ-5D is a widely used QoL measurement in both research and clinical settings.^20^ The questionnaire covers five dimensions, each with a 3-level response (no problem, slight problem, and extreme problem): (i) mobility, (ii) self-care, (iii) usual activities, (iv) pain and discomfort, and (v) anxiety and depression. A second part of the questionnaire consists of the EQ-VAS which is rating of overall health, calibrated from ‘the worst health you can imagine’ to ‘the best health you can imagine’ (scored 0–100).

WHO-5 is a worldwide used questionnaire designed to measure subjective psychological well-being and has been shown to have good clinical validity.^21,24^ It comprises five statements, which participants rate according to a scale (in relation to the past two weeks); 5–0 (all of the time—at no time). The total raw score, ranging from 0 to 25, is multiplied by 4 to give the final WHO-5 index score, with 0 representing the worst imaginable well-being and 100 representing the best imaginable well-being.

### Statistical analysis

No separate sample size calculation for this substudy was performed. The main analysis in this subgroup reporting QoL was performed applying the ITT principle, including all eligible patients who were enrolled and underwent randomization. Secondary analyses include on-treatment analyses where patients in the beta-blocker group were considered non-adherent and were censored if no beta-blocker use was reported at follow-up visits. Patients randomized to no beta-blocker treatment were considered non-adherent and were censored if beta-blocker use was reported at follow-up visits.

To explore possible heterogeneity in the treatment effect, we performed prespecified subgroup analyses of the main outcome by stratification based on sex, age, hypertension, diabetes, previous AMI, infarct type, complete or non-complete revascularization, renal function, and beta-blocker use at baseline.

Continuous variables are presented as medians and quartiles (Q1, Q3) and categorical variables are described in numbers with corresponding percentages. The dimensions from EQ-5D and WHO-5 questionnaires, for each follow up, are presented as median with corresponding IQR and numbers of missing values.

Main outcome was EQ-5D index with beta-blocker status as main exposure. The EQ-5D index score is the converted single weighted index score based on the entire questionnaire. This index score was based on the Swedish population norm.^25^ Wilcoxon rank sum test and ordinal regression analyses (adjusted for age and sex) were used to compare the two groups at each follow-up time point. Missing data were regarded as missing completely at random, no imputation was applied.

Data processing and analysis were performed in R version 4.2.2 (R foundation for Statistical Computing, Vienna, Austria).

## Results

### Patients and follow-up

**Figure 1:**
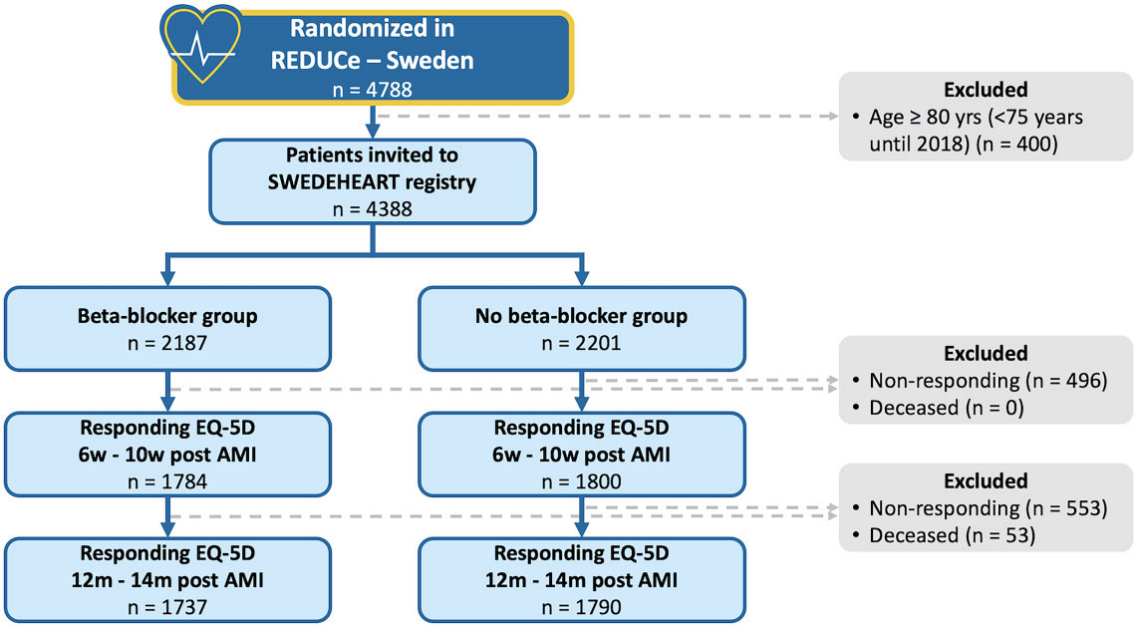
Flow chart for patients within set age limit who were invited to follow-up 1 and 2 (6–10 weeks and 11–13 moths) and reported EQ-5D.

From September 2017 to the end of enrollment in May 2023, a total of 5020 patients underwent randomization in the REDUCE-MI trial,^14^ with 4788 patients (95.4%) from Sweden. A total of 4388 (91.8%) patients <80 years of age (<75 years until 2018) were invited to the SWEDEHEART registry follow-up. Data were extracted in November 2023. A total of 4080 (92.9%) patients responded to at least one of the EQ-5D questionnaires where 3584 (87.8%) patients reported EQ-5D results after 6–10 weeks, and 3527 (86.5%) after 11–13 months after index hospitalization, either partly or completely (*Figure 1*). Overall, 0 and 53 patients died before follow-up 1 and 2, respectively.

The baseline characteristics (*Table 1*) were well balanced between the groups and similar to the baseline data of all participants in the main trial.^14^ Median age was 64 years and 21% were female, 44% had hypertension, 13% had diabetes mellitus, 6% had a prior AMI, and <1% prior heart failure. At time of hospital admission, 10% were on treatment with beta-blockers.

**Table 1:** Baseline characteristics

Variable	Overall, *N* = 4080^1^	No beta-blocker, *N* = 2057^1^	Beta-blocker, *N* = 2023^1^	Difference^2^	95% CI^2,3^
**Demography**					
Median age (IQR)—year	64 (57, 71)	64 (57, 71)	64 (57, 71)	0.01	−0.05, 0.07
Female sex, no (%)	884 (21.7%)	450 (21.9%)	434 (21.5%)	0.01	−0.05, 0.07
**Risk factors**					
Current smoker, no (%)	846 (21.0%)	455 (22.4%)	391 (19.6%)	0.07	0.01, 0.13
Hypertension, no (%)	1795 (44.0%)	906 (44.1%)	889 (44.0%)	0.00	−0.06, 0.06
Diabetes, no (%)	531 (13.0%)	273 (13.3%)	258 (12.8%)	0.02	−0.05, 0.08
**Prior cardiovascular disease**					
Prior myocardial infarctions, no (%)	246 (6.0%)	133 (6.5%)	113 (5.6%)	0.04	−0.02, 0.10
Prior PCI, no (%)	224 (5.5%)	125 (6.1%)	99 (4.9%)	0.05	−0.01, 0.11
Prior CABG, no (%)	51 (1.3%)	25 (1.2%)	26 (1.3%)	-0.01	−0.07, 0.06
Prior stroke, no (%)	82 (2.0%)	45 (2.2%)	37 (1.8%)	0.03	−0.04, 0.09
Prior heart failure, no (%)	24 (0.6%)	17 (0.8%)	7 (0.3%)	0.06	0.00, 0.13
**Presentation characteristics**					
Chest pain as main symptom, no (%)	3945 (96.7%)	1986 (96.5%)	1959 (96.8%)	−0.02	−0.08, 0.05
CPR before hospital, no (%)	16 (0.4%)	10 (0.5%)	6 (0.3%)	0.03	−0.03, 0.09
Heart rate, median (IQR)	74 (64, 84)	73 (64, 84)	74 (64, 85)	−0.01	−0.07, 0.06
Systolic blood pressure, median (IQR)	152 (137, 170)	152 (137, 170)	152 (136, 170)	−0.02	−0.08, 0.05
On beta-blocker treatment, no (%)	398 (9.9%)	213 (10.5%)	185 (9.3%)	0.04	−0.02, 0.10
**In-hospital course**					
Coronary angiography				0.03	−0.04, 0.09
1-vessel disease, no (%)	2336 (57.3%)	1166 (56.7%)	1170 (57.9%)		
2-vessel disease, no (%)	1082 (26.6%)	552 (26.9%)	530 (26.3%)		
LM or 3-vessel disease, no (%)	616 (15.1%)	317 (15.4%)	299 (14.8%)		
Other	40 (1.0%)	20 (1.0%)	20 (1.0%)		
**Medication at discharge**					
Aspirin, no (%)	3987 (97.7%)	2008 (97.6%)	1979 (97.8%)	0.01	−0.05, 0.08
P2Y12-rec blockade, no (%)	3946 (96.7%)	1986 (96.5%)	1960 (96.9%)	0.02	−0.04, 0.08
Calcium antagonists, no (%)	714 (17.5%)	389 (18.9%)	325 (16.1%)	0.07	0.01, 0.14
Beta-blockers, no (%)	2122 (52.0%)	187 (9.1%)	1935 (95.7%)	3.5	3.4, 3.6
ACE inhibitors or ARB, no (%)	3285 (80.5%)	1678 (81.6%)	1607 (79.4%)	0.05	−0.01, 0.11
Statins, no (%)	4031 (98.8%)	2023 (98.4%)	2008 (99.3%)	0.08	0.02, 0.14
Diuretics, no (%)	292 (7.2%)	139 (6.8%)	153 (7.6%)	0.03	−0.03, 0.09

1 Median (IQR); *n* (%).

2 Standardized mean difference.

3 CI = Confidence interval.

IQR, interquartile range; PCI, percutaneous coronary intervention; CABG, coronary artery by-pass grafting; CPR, cardiopulmonary resuscitation, LM, left main, ACEI; angiotensin-converting-enzyme inhibitors; ARB, angiotensin receptor blockers.

From July 2018 to the end of June 2022, a total of 806 patients were included in the WHO-5 subgroup and had similar baseline characteristics (Supplementary material online, *Figure S1*, Supplementary material online, *Table S1*).

### Treatment adherence

Of the 4080 eligible patients, 2023 patients (49.6%) were randomized to beta-blocker treatment and 2057 patients (50.4%) to no-beta-blocker treatment. Using the definition of protocol adherence on data derived from the SWEDEHEART follow-up visits, 1693 patients (90.3%) and 1470 (81.8%) in the beta-blocker group were still taking beta-blockers after 6–10 weeks and 11–13 months, respectively. In the no-beta-blocker group, 214 (11.3%) and 263 (14.2%) were prescribed beta-blockers after 6–10 weeks and after 11–13 months, respectively.

### Outcomes

**Figure 2:**
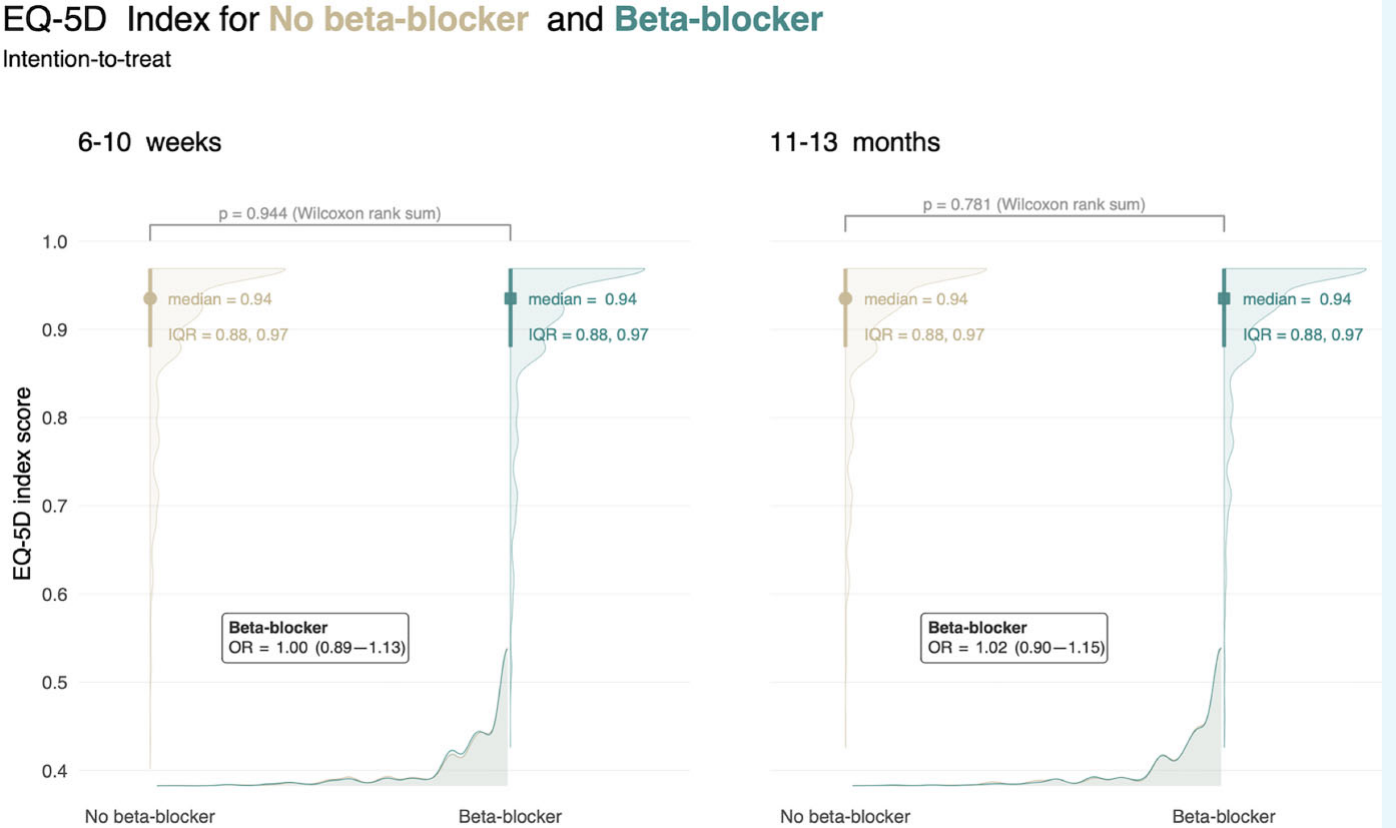
Main outcome. EQ-5D index score analysed according to intention-to-treat at 6–10 weeks and 11–13 months after acute myocardial infarction. Difference in medians calculated with Wilcoxon rank sum test with corresponding *P*-value. Odds ratio (OR) analysed with ordinal regression method adjusting for age and sex. IQR denotes interquartile range, OR with confidence intervals in brackets.

In the ITT analysis, the main outcome, median EQ-5D index score, was 0.94 (IQR 0.88, 0.97) in the beta-blocker group and 0.94 (IQR 0.88, 0.97) in the no-beta-blocker group 6–10 weeks after AMI (follow-up 1), Odds ratio (OR) 1.00 (95% CI 0.89–1.13, *P* > 0.9). After 11–13 months (follow-up 2), the results were unchanged, 0.94 (IQR 0.88, 0.97) in the beta-blocker group and 0.94 (IQR 0.88, 0.97) in the no-beta-blocker group, OR 1.02 (95% CI 0.90–1.15, *P* = 0.8) (*Figure 2*). The on-treatment analyses yielded similar results as the ITT analysis. The main outcome, the median EQ-5D index score, was 0.94 (IQR 0.88, 0.97) in the beta-blocker group and 0.94 (IQR 0.88, 0.97) in the no-beta-blocker group 6–10 weeks after AMI, OR 0.92 (95% CI 0.81–1.04, *P* = 0.2). After 11–13 months, results remained robust, 0.94 (IQR 0.88, 0.97) in the beta-blocker group and 0.94 (IQR 0.90, 0.97) in the no-beta-blocker group, OR 1.01 (95% CI 0.89–1.15, *P* = 0.8).

**Figure 3:**
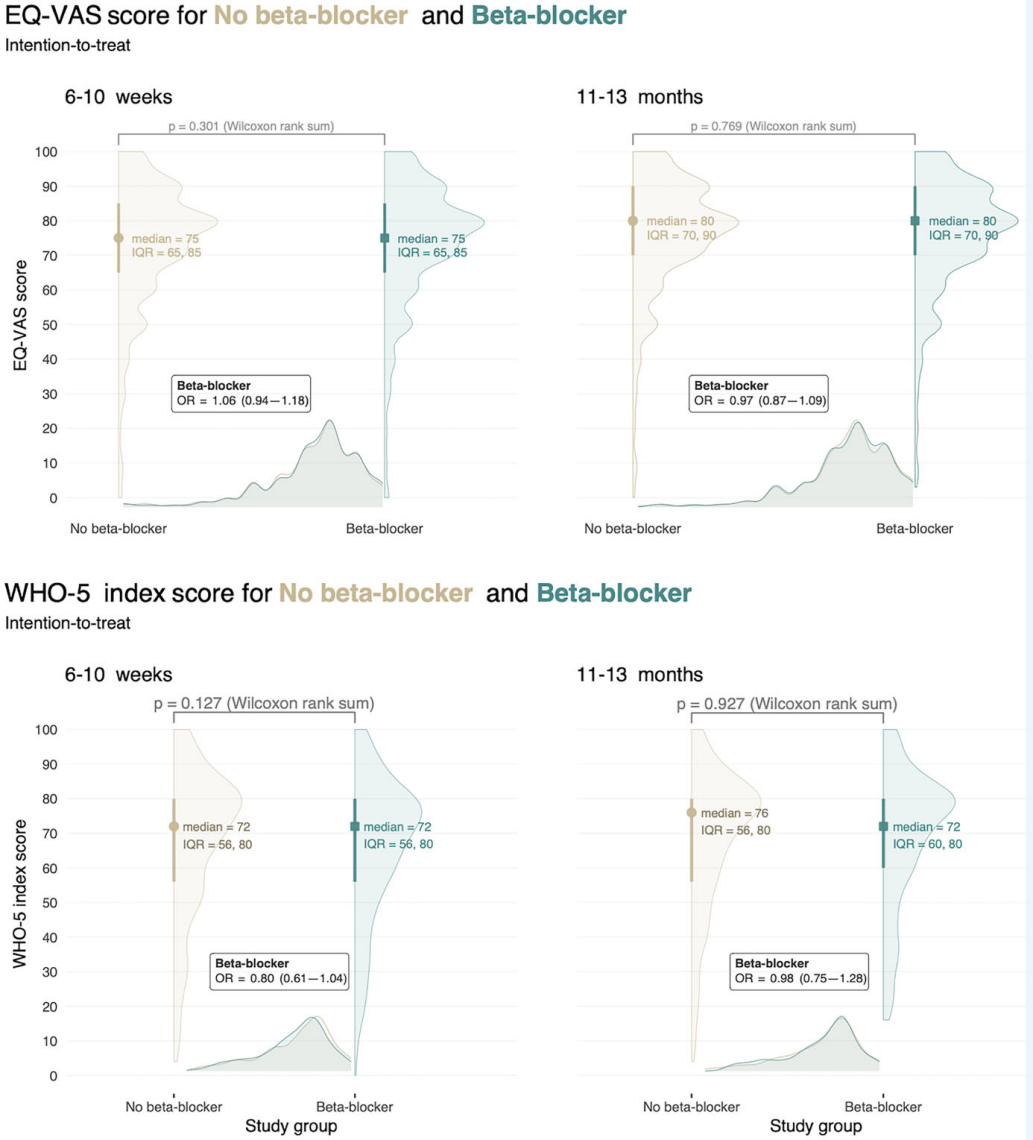
Secondary outcomes. EQ VAS score and WHO-5 index score analysed according to intention-to-treat at 6–10 weeks and 11–13 months after acute myocardial infarction. Difference in medians calculated with Wilcoxon rank sum test with corresponding *P*-value. Odds ratio (OR) analysed with ordinal regression method adjusting for age and sex. IQR denotes interquartile range, OR odds ratio with confidence intervals in brackets.

The secondary outcome, median EQ-VAS score was 75 (IQR 65, 85) in the beta-blocker group and 75 (IQR 65, 85) in the no-beta-blocker group 6–10 weeks after AMI, OR 1.06 (95% CI 0.94–1.18, *P* = 0.4). After 11–13 months, EQ-VAS results remained similar between groups at a slightly higher level, 80 (IQR 70, 90) in the beta-blocker group, 80 (IQR 70, 90) in the no-beta-blocker group, OR 0.97 (95% CI 0.87–1.09, *P* = 0.7) (*Figure 3*). Median WHO-5 index score, was 72 (IQR 56, 80) in the beta-blocker group and 72 (IQR 56, 80) in the no-beta-blocker group 6–10 weeks after AMI, OR 0.80 (95% CI 0.61–1.04, *P* = 0.09). After 11–13 months, median WHO-5 index score was 72 (IQR 60, 80) in the beta-blocker group and 76 (IQR 56, 80) in the no-beta-blocker group, OR 0.98 (95% CI 0.75–1.28, *P* = 0.88) (*Figure 3*).

### Subgroup analyses

**Figure 4:**
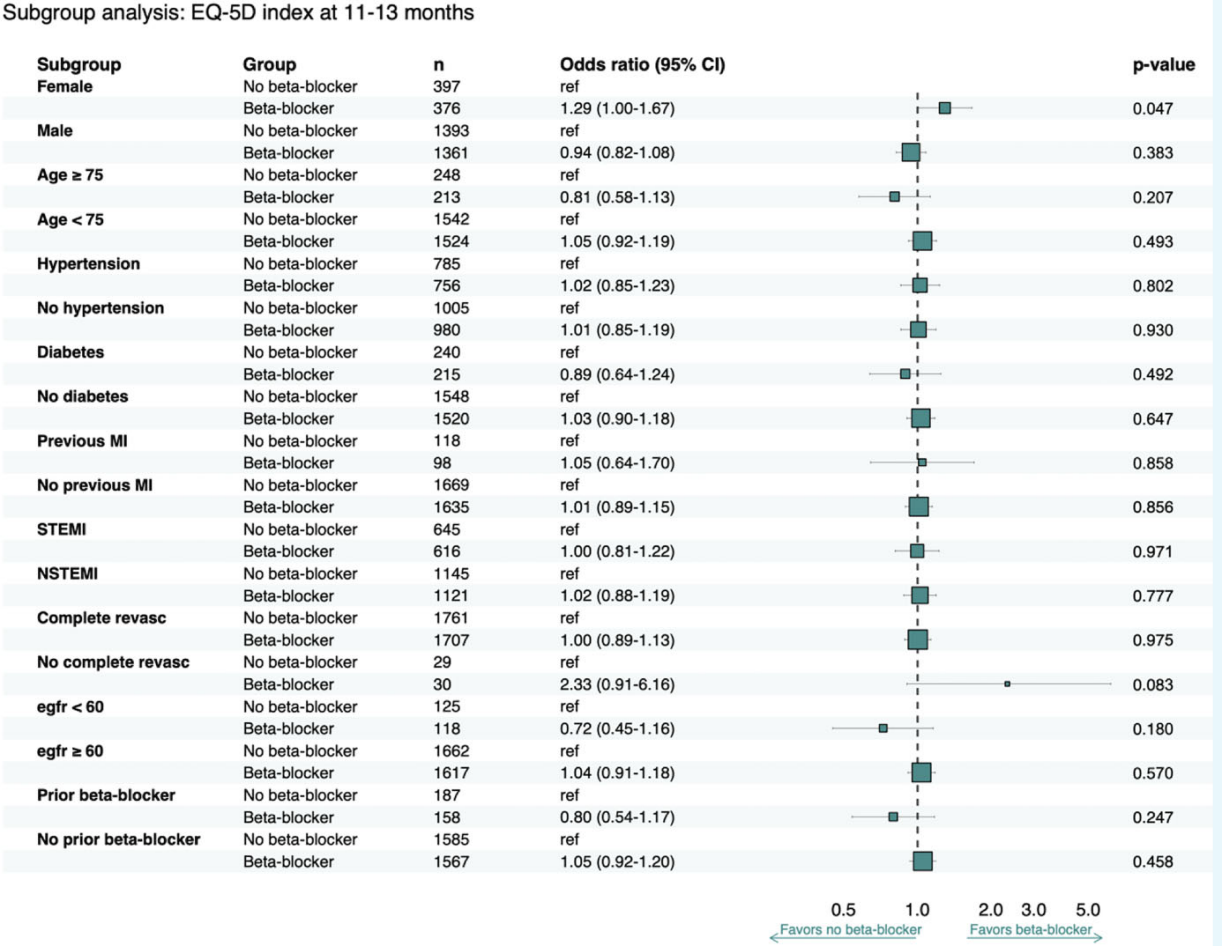
Subgroup analysis presented as a Forest Plot. EQ-5D index score stratified into subgroups according to intention-to-treat analysis at 11–13 months after acute myocardial infarction.

Stratification according to aforementioned subgroups indicated similar effects regarding the main outcome, i.e. EQ-5D index score, both at 6–10 week and 11–13 months follow-up. The exception was in the subgroup divided by sex where females randomized to beta-blockers had a marginally but significantly higher EQ-5D index score after 11–13 months compared to females in the no-beta-blocker group, OR 1.29 (1.00–1.67; *P* = 0.047) (*Figure 4*, Supplementary material online, *Figure S2*).

## Discussion

In the REDUCE-AMI study, long-term beta-blocker use in patients after AMI and preserved LVEF demonstrated neither effect on cardiovascular outcomes nor any significant clinical safety concerns.^14^ In the present study, we did not find any effect of initiation of long-term beta-blocker therapy on self-reported QoL, general health status, or well-being when compared with no-beta-blocker treatment. These findings remained consistent over both short (6–10 weeks) and long-term (11–13-month) follow-up after AMI, regardless of ITT or on-treatment analyses and were robust across relevant subgroups.

Since this is the first randomized trial evaluating long-term effects of beta-blockers on QoL and well-being in patients with AMI and preserved LVEF, we do not have any contemporary studies to compare our results with. The landmark trials were performed in an era prior to early reperfusion strategies and use of effective secondary prevention methods and differ from our trial in two key aspects: First, the majority of individuals at the time suffered large AMIs commonly leading to subsequent heart failure, arrhythmias, or death. Second, treatment schemes and clinical practice included rigorous titration of beta-blockers to a maximum tolerated dose, often a target dose of 200 mg metoprolol or equivalent, while our study encouraged treating physicians to aim for at least 100 mg and 5 mg Metoprolol and Bisoprolol respectively, most likely leading to lower doses compared with landmark trials. Some observational studies from the following decades found beta-blockers associated with symptoms of depression and sexual dysfunction, factors that are known to affect QoL, while other studies could not confirm these association.^9,11,26,27^

Overall, guidelines and clinical practice retained a strong treatment recommendation for beta-blockers after AMI based on the belief of clinical efficacy, while accepting remaining controversy of potential adverse effects with regard to QoL.^1^ In our contemporary study, patients had preserved ejection fraction and underwent early revascularization with optimal secondary prevention therapy leading to low event rates and excellent survival regardless of beta-blocker treatment.^14^ Thus, the utility of our neutral QoL assessments need to be appreciated in context to the substantially evolved underlying patient population and clinical practice since the landmark trials.

### Trial results in relation to earlier evidence

The neutral effect on self-reported QoL may rely on several aspects. We found that contemporary AMI patients have a swift recovery and good prognosis without major impact on QoL regardless of beta-blocker use which aligns well with previous Qol estimates from the general Swedish population.^28^ Before the results of the REDUCE-AMI study were available, and to assess representativeness of the trial population, we conducted an observational benchmark study emulating the trial within the SWEDEHEART registry using data from 10 926 patients eligible for inclusion over the 7-year period prior to the REDUCE-AMI trial.^29^ In comparison, the REDUCE trial included 4788 patients from SWEDEHEART over the subsequent 6.5 years, despite a lower incidence of myocardial infarction during this time. This suggests that more than 50% of all eligible patients were included in the trial. Moreover, both baseline characteristics and event rates were similar in the trial and in the observational study, indicating a high degree of representativeness of the REDUCE-AMI trial.

Symptoms of angina are common after AMI and may influence QoL. Beta-blockers have anti-anginal effects well-established for decades and are recommended as first line therapy in guidelines.^30^ In the main REDUCE-AMI trial, we analysed angina symptoms routinely obtained during the SWEDEHEART follow-up without detecting any difference between the randomized groups within one year^14^ (Supplementary material online, *Figures S10* and *S11*) in alignment with the neutral findings of the present analysis. However, this lack of difference may be partly explained by crossovers or use of other anti-anginal drugs such as nitrates or calcium-channel blockers. Unfortunately, at this stage, we do not have data from the prescribed drug registry available to further explore that possibility.

In alignment with the pragmatic design of the REDUCE-AMI trial, dosing of beta-blockers was at the discretion of the treating physician which most likely led to considerably lower doses than in the aforementioned landmark trials. Unfortunately, at this time-point, we do not have data available on prescribed doses. However, a recent large observational study from the SWEDEHEART registry in 36 000 individuals compared patients after AMI discharged with ≥50% of the target beta-blocker dose compared with patients at <50% of the target beta-blocker dose. In this unselected population with two thirds of patients treated with low dose beta-blockers, there was no difference in QoL outcomes between high and low beta-blocker dose.^31^ Lower dosing of beta-blockers has also been reported in observational studies from other healthcare systems,^32–34^ indicating a good alignment with clinical practice in the present study. The underlying reasons for lower dosing are not fully understood. Treating physicians today may not aim for higher doses of beta-blockers due to the risk of unwanted side effects of bradycardia or hypotension in the light of unproven clinical efficacy. Lower patient acceptance of subjective symptoms, including fatigue, mood changes, or sexual dysfunction may also play a larger role today. The signal of benefit in the higher EQ-5D index score after 11–13 months in women on beta-blockers compared with no beta-blockers is of uncertain significance and should be interpreted with great caution. Sex-specific QoL results from previous studies are overall scarce, and if reported at all, inconsistent. If such adverse effects of beta-blockers exist must therefore be studied in future trials specifically addressing this question.

### Impact of trial design and QoL instruments

The primary objective of the REDUCE-AMI trial was to test the strategy of routine initiation of beta-blockers to a broad population of MI patients fulfilling the eligibility criteria in contrast to testing the effects of beta-blockers on eligible individuals. In a pragmatic register-based RCT approach, as used in our trial, utilizing streamlined trial procedures with data obtained within routine practice is essential for the conducts feasibility but comes at the price of less detail.^35^ In our case, e.g. on-going treatments including beta-blockers are obtained during the SWEDEHEART follow-up but no doses or reasons for treatment changes are reported. We used the EQ-5D and WHO-5 questionnaires, which are both widely used and well validated in clinical settings,^20,21,24^ also for practical reasons as the EQ-5D is obtained during routine post-AMI follow-up in Sweden.^18^ While the EQ-5D is appreciated in clinical context as being simple and as relevant as possible to all respondents, it has also been criticized for simplification of the outcome and generic classification of health-related QoL.^36^ Obviously, data-rich standardized questionnaires that delve further into health aspects related to QoL in comparison to the EQ-5D would have been preferable but were deemed unfeasible. Overall, we believe that the combined use of EQ-5D, EQ-VAS, and WHO-5 including separated evaluation of each dimension, derived from a large number of participants was an adequate pragmatic choice and should yield a reliable estimate of the patients’ general QoL. In the near future, other large, on-going trials examining long-term treatment with beta-blockers in similar AMI patients aim to report Qol endpoints obtained with similar instruments which will clarify remaining uncertainties.^37,38^

### Clinical implications

The neutral results of this substudy on QoL and well-being are well in line with the neutral finding of the main analysis with regard to cardiovascular outcomes, mortality, and safety. It appears that routine beta-blocker treatment does neither do much good nor cause obvious harm. Therefore, it seems reasonable to be more restrictive in initiating beta-blocker treatment in similar AMI patients as in our trial. Supportive of this notion is the fact that drug non-adherence overall increases with polypharmacy, in particular with beta-blockers,^39^ which underscores the importance to restrict preventive measure to treatments with proven efficacy. Moreover, health economic considerations apply as well. Despite a relatively low cost of beta-blockers by itself, expenditure for the health care system accumulate with regard to the high incidence of MI, and the fact that historical treatment after AMI was for long-term use which leads to considerable accumulated costs over time. Potential savings for the health care systems have been described previously for other AMI related therapies with limited effect on cardiovascular outcomes,^40,41^ and are planned for the REDUCE-AMI trial as well.

The strength of our trial lies in the large sample size from a randomized trial performed in a pragmatic fashion incorporating routine care processes from clinical practice. Overall, as a substudy not involving all randomized individuals, the available data becomes more observational in nature and should be interpreted as such. In addition to limitations with regard to study design, study population, treatment preferences of treating physicians, and applied QoL instruments described above, a major limitation for this substudy, was the open-label design of the REDUCE-AMI trial. Patients were informed about the different possible side effects of beta-blockers before accepting participation, and patients generally knew their treatment assignment before reporting any of the questionnaires. Moreover, not all REDUCE-AMI participants were eligible or for other reasons enrolled in this substudy which may led to a more selected population reducing external validity. However, the treatment groups were well balanced with regard to baseline characteristics and did not significantly differ from the overall population, indicating a representative patient enrollment. Finally, at this stage, we lack information during follow-up on some beta-blocker related adverse events or new treatment indications, and socioeconomic status which may have impacted on the on-treatment results.

## Conclusion

Among patients after AMI with preserved LVEF, self-reported QoL and well-being was not significantly different in individuals randomized to routine long-term beta-blocker therapy as compared with individuals with no beta-blocker use. These results appear consistent regardless of adherence to randomized treatment and across subgroups which emphasizes the need for a careful individual risk-benefit evaluation prior to initiation of beta-blocker treatment.

## Supplementary Material

pvae062_Supplemental_File

## Data Availability

The data underlying this article cannot be shared publicly due to the General Data Protection Regulation (2016/679). The data will be shared on reasonable request to the corresponding author.

## Notes

**Conflict of interest:** Dr Hofmann reports lecture fees to institution from AstraZeneca, Pfizer, and BMS. Dr Held reports consulting fees from AstraZeneca, Boehringer Ingelheim, NovoNordisk, CoalaLife, and Pharmacosmos. Dr Held also reports lecture fees from Amarin and Bayer. Dr Jernberg reports institutional grants from MSD and consulting fees from Amgen. Dr Ravn-Fischer reports lecture fees from Amarin, Amgen, AstraZeneca, BMS, Boehringer, Janssen, Novartis, NovoNordisk, Orion Pharma, Pfizer, and Sanofi as well as advisory board fees from Amarin, Novartis, NovoNordisk, and Sanofi. Dr Alfredsson report lecture fees from Novartis, Orion Pharma, Boehringer Ingelheim, and AstraZeneca as well as advisory board fees from Novartis and AstraZeneca. Dr Lauermann reports advisory board fees from Chiesi and Bayer. Dr Karlström report lecture fees from AstraZeneca and Boehringer Ingelheim as well as advisory board fees from AstraZeneca and Novartis.
