# Supplementary material for: Effects of beta-blockers on quality of life and well-being in patients with myocardial infarction and preserved left ventricular function—a prespecified substudy from REDUCE-AMI
Source: Eur Heart J Cardiovasc Pharmacother. 2024 Sep 1;10(8):708–18. doi: 10.1093/ehjcvp/pvae062 (PMC11724137; doi:10.1093/ehjcvp/pvae062)
Supplement: pvae062_Supplemental_File [file pvae062_supplemental_file.docx]

Supplementary Appendix to:

**Effects of Beta-Blockers on Quality of Life and Well-being in Patients with Myocardial Infarction and Preserved Left Ventricular Function**

- **a prespecified substudy from REDUCE-AMI**

*Katarina Mars, M.D.,^1^ Sophia Humphries, M.Sc., Ph.D., ^2^ Philip Leissner, M.Sc., ^2^ Martin Jonsson, Ph.D., ^1^ Patric Karlström, M.D., Ph.D., ^3^ Jörg Lauermann, M.D, Ph.D.,³ Joakim Alfredsson, M.D., Ph.D., ^4^ Thomas Kellerth, M.D., ^5^ Annica Ravn-Fischer, M.D., Ph.D., ^6^ David Erlinge, M.D., Ph.D., ^7^ Bertil Lindahl, M.D., Ph.D.,^9, 10^ Troels Yndigegn, M.D., ^7^ Tomas Jernberg, M.D., Ph.D.,^8^ Claes Held, M.D., Ph.D.,^9,10^ Erik M.G. Olsson, Ph.D.,^2^ and Robin Hofmann, M.D., Ph.D.^1^*

1. Department of Clinical Science and Education, Division of Cardiology, Karolinska Institutet, Södersjukhuset, Stockholm, Sweden.
2. Department of Women’s and Children’s Health, Uppsala University, Uppsala, Sweden.
3. Department of Internal Medicine, Ryhov County Hospital, Jönköping Sweden.
4. Department of Cardiology and Department of Health, Medicine and Caring Sciences, Linköping University, Linköping, Sweden.
5. Division of Cardiology and Emergency medicine, Centralsjukhuset Karlstad, Karlstad, Sweden, Sweden.
6. Department of Cardiology, Sahlgrenska University Hospital, Institute of Medicine, Department of Molecular and Clinical Medicine, Sahlgrenska Academy University of Gothenburg, Gothenburg, Sweden.
7. Department of Cardiology, Clinical Sciences, Lund University, Skane University Hospital, Lund, Sweden.
8. Department of Clinical Sciences, Danderyd Hospital, Karolinska Institutet, Stockholm, Sweden.
9. Department of Medical Sciences, Cardiology, Uppsala University, Uppsala, Sweden.
10. Uppsala Clinical Research Center, Uppsala University, Uppsala, Sweden.

**Table of Contents**

Study Personnel p. 3

List of investigators by participating center, EQ-5D p. 3

List of investigators by participating center, RQoL p. 6

Detailed description of study population and data collection for WHO-5 p. 7

Tables and Figures p. 8 - 17

Table S1: Baseline WHO-5, ITT p. 8

Table S2: Baseline EQ-5D, on-treatment p. 9

Table S3: EQ-5D ITT, FU 1 p. 11

Table S4: EQ-5D, ITT, FU 2 p. 12

Table S5: EQ-5D, on-treatment, FU 1 p. 13

Table S6: EQ-5D, on-treatment, FU 2 p. 14

Table S7: Ordinal regression model, EQ-5D/VAS, ITT p. 15

Figure S1: Flow-chart WHO-5 p. 16

Figure S2: Subgroup analysis EQ-5D, FU1 p. 17

**Study Personnel**

**List of Investigators by participating center, EQ-5D**

Troels Yndigegn, Skåne University Hospital, Lund ((National Lead Investigator)

1. Anders Holmdahl, Anna Hammar, Gösta Westerberg, Josefine Orsjö, Lars Andersson, Malin Rylander, Peter Karlsson, and Rikard Roupe, Department of Medicine, Alingsås Hospital, Alingsås
2. Alexander Wasmuth, Hans Tygesen, Haval Mustafa, Ilona Yevno, Susanne Johansson, Tomas Jonson, and Marcus Lycksell, Department of Medicine, Södra Älvsborg Hospital, Borås
3. Ann Samnegård, Catrine Edström-Plüss, Fadi Jokhaji, Fredrik Wallentin, Mattias Ekström, Mattias Törnerud, Claes Hofmann-Bang, Johannes Aspberg, Patrik Hjalmarsson, Pia Lundman, Sara Aspberg, Tomas Jernberg, Department of Cardiology, Danderyd Hospital, Danderyd
4. Frida Bergenholm, Peter Lindholm, Melinda Csegedi, Department of Medicine, Eksjö Hospital, Eksjö
5. Hanna Alwan, Kave Keshavarz, Linda Ärlehag, Mehmet Hamid, Mohamed Alharis Radawe, Nabeel Maarouf Mansour, Ninve Palo, Oscar Eriksson, Qassim Awad, Georgios Matthaiou, Department of Medicine, Mälarsjukhuset, Eskilstuna
6. Carl Bergman, Carl Gustafsson, Greger Tysk, Julia Söderberg, Kristina Carlsson, Åsa Drakesby, Department of Cardiology, Falu Hospital, Falun
7. Kristina Sumske, Cornelia Varadan, Halland Hospital, Halmstad
8. Carl-David Dolata, Ole Wagner, Henrik Wagner, Department of Cardiology, Helsingborg Hospital, Helsingborg
9. Antheia Kissopoulou, Jörg Lauermann, Karim Abdulhadi, Marija Jakovlevski Hedbäck, Neshro Barmano, Tomasz Zwierzchlewski Patric Karlström, Department of Cardiology, Ryhov Hospital, Jönköping
10. Erik Hoffman, Moa Simonsson, Robert Edfors, Stefan Lind, Stina Smetana, Susanna Boquist, Department of Cardiology, Karolinska University Hospital, Solna
11. Marie Björkenstam, Maria Wideqvist, Sara Bentzel, Annica Ravn-Fischer, Department of Cardiology, Sahlgrenska University Hospital, Göteborg
12. Aferdita llapaj Hetemi, Georgios Mourtzinis, Janet Moodh, Lars Klintberg, Martin Risenfors, Pär Parén, Pia Eriksson de Luca, Pia Linton Dahlöf, Therese Westberg, Ulf Axelsson, Fredrik Melchior, Department of Cardiology, Sahlgrenska University Hospital Mölndal, Göteborg
13. Charlotte Magnusson, Per Lundberg, Maria Wideqvist, Department of Cardiology, Sahlgrenska University Hospital Östra, Göteborg
14. Dimitri Matan, Göran Kennebäck, Karin Ljung, Layth Aladellie, Liyew Desta, Moayad Al-Azzawy, Staffan Eliasson, Stefan Lind, Thomas Flodin, Karolina Szummer, Department of Cardiology, Karolinska University Hospital, Huddinge
15. Anders Engström, David Olsson, Anja Fagerström, Department of Medicine, Kalmar Hospital, Kalmar
16. Anna Lenberg, Birgit Echeverri, Karl-Anders Nilsson, Rakel Lindqvist-Rosengren, Viktoria Hermansson, Ola Hallén, Thomas Kellerth, Urban Haaga, Department of Cardiology and Emergency Medicine, Centralsjukhuset Karlstad, Karlstad
17. Bodil Dahl, Björn Karlqvist, Diodor Cojucaru, Hannes Lynner, Marian Tudor, Per-Anders Wiklund, Raluca Jumatate, Helena Wikström, Department of Medicine, Kristianstad Hospital, Kristianstad
18. Gunvor Finnas, Johannes Strandberg Anna Alestig, Department of Medicine, Kungälv Hospital, Kungälv
19. Claudia Backlund, Gull-Britt Eriksson, Susanne Hahne, Ali Muttar, Department of Medicine, Västmanland Hospital, Köping
20. Karin Lundgren, Magnus Peterson, Sofia Svensson, Ulrika Pettersson, Hanna Österman, Department of Medicine, Skaraborg Hospital, Lidköping
21. Anna Holm, Eva Swahn, Maria Eriksson, Mats Pettersson, Mona Börjesson, Sofia Sederholm Lawesson, Ted Cabreira, Joakim Alfredsson, Department of Cardiology, Linköping University Hospital, Linköping
22. Asos Mirkhan, Carina Nilsson, Gustav Lund, Malin Kihlberg, Viveca Ritsinger, Department of Medicine, Ljungby Hospital, Ljungby
23. Anna Duckert, Anneli Olsson, Arash Mokhtari, Erik Rydberg, Gunilla Brolin, Jakob Lundgren, Josef Dankiewicz, Lotta Cinthio, Enisa Durakovic, Nazim Isma, Troels Yndigegn, David Erlinge, Department of Cardiology, Skåne University Hospital, Lund
24. Enisa Durakovic, Robert Jablonowski, Troels Yndigegn, Klas Persson, Department of Cardiology, Skåne University Hospital, Malmö
25. Solveig Östberg, Sascha Wagner, Department of Medicine, Mora Hospital, Mora
26. Mia Grändås, Noorullah Moosawi, Åsa Törnqvist, Department of Medicine, Motala Hospital, Motala
27. Malgorzata Pierscinska-Jedra, Maria Pettersson, Ulla Sahlen, Christofer Digerfeldt, Department of Cardiology, Vrinnevi Hospital, Norrköping
28. Annelie Johansson, Emil Grönlund, Kamaran Mohammed, Marianne Erlandsson, Michael Eriksson, Göran Arstad, Department of Cardiology, Capio S:t Görans Sjukhus, Stockholm
29. Anders Hallenberg, Carina Hägglund, Gunnar Boberg, Lis Kohlström, Luwam Habtemariam, Marie Jernvald, Marcus Arvidsson, Mats Frick, Mia Henning, Runa Sundelin, Katarina Mars, Robin Hofmann, Department of Cardiology, Södersjukhuset, Stockholm
30. Maria Stolt-Toremark, Mikael Olsson, Lisa Brandin, Department of Cardiology, Skaraborg Hospital, Skövde
31. Cecilia Boltemo Nilsson, Philip Morsing, Staffan Stålnacke Philip Morsing, Department of Cardiology, Sunderbyn Hospital, Sunderbyn
32. Erik Benedik, Gabriel Fuchs, Jens Hällgren, Lina Mattsson, Louise Persson, Pernilla Ulander, Sara Själander, Department of Medicine, Sundsvall Hospital, Sundsvall
33. Fredrik Valham, Per Ottander, Rikard Hedelin, Therese Andersson, Ellinor Bergdahl, Department of Cardiology, Norrland University Hospital, Umeå
34. Kasper Andersen, Anton Gard, Axel Åkerblom, Christina Björklund, Helena Wall, Kai Eggers, Louise Robertsson, Maciej Olszowka, Maria Andreasson, Johan Sundström, Claes Held, Bertil Lindahl, Department of Cardiology, Uppsala University Hospital, Uppsala
35. Ann-Lis Wännman, Angela Synnerö, Carina Gustavsson, Lisbeth Nilsson, Torbjörn Vik, Department of Medicine, Halland Hospital, Varberg
36. Carina Andersson, Marika Lundqvist, Charlotta Myllylä, Saman Saidi-Seresht, Department of Medicine, Västmanland Hospital, Västerås
37. Anna Mattsson, Anna-Sara Sundelin, Emanuel Frimodig, Karin Johansson, Karin Sjöö, Margit Quist, Robin Gustafsson, Thomas Kellerth, Torbjörn Kalm, Yakak Akyuz, Ole Fröbert, Department of Cardiology, Örebro University Hospital, Örebro
38. Eva Höglund, Jacob Vackdahl, Maria Kjesbo, Ulf Kajermo, Anders Ulvestam, Department of Medicine, Östersund Hospital, Östersund

**List of Investigator by participating center, RQoL**

1. Lena Bergvall-Henriksson, Liselott Persson, and Tomas Jernberg, Department of Cardiology, Danderyd Hospital, Danderyd
2. Annika Koch, Jessica Samuelsson, Neshro Barmano, Jörg Lauermann, and Patric Karlström, Department of Cardiology, Ryhov Hospital, Jönköping
3. Maria Eriksson, and Joakim Alfredsson, Department of Cardiology, Linköping University Hospital, Linköping
4. Anna Duckert, and Troels Yndigegn, Department of Cardiology, Skåne University Hospital, Lund
5. Lis Kohlström, Mia Henning, Runa Sundelin, Robin Hofmann, and Katarina Mars, Department of Cardiology, Södersjukhuset, Stockholm
6. Christina Björklund, Helena Gårdesten-Wall, Sophia Humphries, Philip Leissner, Claes Held, and Erik Olsson, Department of Cardiology, Uppsala University Hospital, Uppsala
7. Carina Andersson, Marika Lundqvist, Charlotta Myllylä, Saman Saidi-Seresht, Department of Medicine, Västmanland Hospital, Västerås
8. Anna Mattsson, Thomas Kellerth, and Emanuel Frimodig, Department of Cardiology, Örebro University Hospital, Örebro

**Detailed description of study population and data collection for WHO-5**

At eight sites in Sweden, we collected additional data on psychological distress, sexual dysfunction, and well-being measures, where well-being data (measured by WHO-5) was included in this article. Patients randomized in REDUCE-AMI trial were, at these sites, invited to take part in the REDUCE-Quality of Life (RQoL) substudy. An added inclusion/exclusion criteria to REDUCE-AMI was the requirement of knowing Swedish at a higher level.

Those willing to participate provided additional consent via a secure online portal, the Uppsala University Psychosocial Care Program (U-CARE) Portal (the Portal) or on paper at the hospital. Self-reported measurements were collected at three time points: baseline (within 0–7 days post-AMI), 6–10 weeks, and 11–14 months post-AMI. Baseline was mainly filled in and collected during hospital stay. For the second and third observation points, participants were automatically sent text message and email notifications at the time for responding to questionnaires. If no response was notified after one week, participants received automatic text message and email reminders. If still no response after another week, the participant was contacted by telephone. For patients participating on paper, forms with the questionnaires were sent out at the observation points and they were reminded in the same way by telephone after two weeks. Paper questionnaire responses were manually recorded into the Portal by research staff.

Questionnaires including measures on psychological distress, sexual dysfunction and well-being were collected at all three time points. Additional data were collected at baseline including information about education level, country of birth, physical activity, smoking status, relationship status, and concerns about unwanted beta-blocker side effects and psychotropic medication use. At both follow-up time points, patients were asked about treatment adherence, and an open question about possible experienced side effects.

A sample size calculation was performed before the start of this substudy. By assuming a standardized mean difference of 0.25 for the outcome measure, and to detect this difference with 80% power at a significance level of 5%, 251 participants in each group would be necessary. To compensate for cross-over and loss-to follow-up, and because of fast inclusion-speed compared to REDUCE-AMI trial, enrolment in the RQoL substudy continued after reaching 502 individuals.

| **Table S1: Baseline WHO-5, ITT** |  |  |  |  |
| --- | --- | --- | --- | --- |
| **Variable** | **No beta-blocker**  N = 397*^1^* | **Beta-blocker**  N = 409*^1^* | **Difference***^2^* | **95% CI***^2,3^* |
| **Demography** |  |  |  |  |
| Median age (IQR) - year | 64 (57, 72) | 66 (57, 73) | -0.09 | -0.23, 0.05 |
| Female sex, no(%) | 85 (21.4%) | 97 (23.7%) | -0.06 | -0.19, 0.08 |
| **Risk Factors** |  |  |  |  |
| Current smoker, no(%) | 75 (19.0%) | 57 (14.1%) | 0.13 | -0.01, 0.27 |
| Hypertension, no(%) | 195 (49.1%) | 205 (50.1%) | 0.02 | -0.12, 0.16 |
| Diabetes, no(%) | 57 (14.4%) | 72 (17.6%) | 0.09 | -0.05, 0.23 |
| **Prior Cardiovascular Disease** |  |  |  |  |
| Previous AMI, no(%) | 30 (7.6%) | 33 (8.1%) | 0.02 | -0.12, 0.16 |
| Previous PCI, no(%) | 29 (7.3%) | 29 (7.1%) | 0.01 | -0.13, 0.15 |
| Previous CABG, no(%) | 8 (2.0%) | 7 (1.7%) | 0.02 | -0.12, 0.16 |
| Previous Stroke, no(%) | 8 (2.0%) | 10 (2.4%) | 0.03 | -0.11, 0.17 |
| Previous heart failure, no(%) | 5 (1.3%) | 3 (0.7%) | 0.05 | -0.09, 0.19 |
| **Presentation Characteristics** |  |  |  |  |
| Chest pain as main symptom, no(%) | 372 (93.7%) | 389 (95.1%) | -0.06 | -0.20, 0.08 |
| CPR before hospital, no(%) | 6 (1.5%) | 2 (0.5%) | 0.10 | -0.04, 0.24 |
| Atrial fibrillation, no(%) | 4 (1.0%) | 3 (0.7%) | 0.03 | -0.11, 0.17 |
| Heart rate, median (IQR), bpm | 73 (63, 86) | 75 (65, 85) | -0.04 | -0.18, 0.10 |
| Systolic blood pressure, median (IQR) | 151 (135, 169) | 150 (132, 167) | 0.03 | -0.10, 0.17 |
| Beta-blockers, no(%) | 56 (14.1%) | 55 (13.4%) | 0.02 | -0.12, 0.16 |
| **In-hospital Course** |  |  |  |  |
| Coronary angiography |  |  | 0.06 | -0.07, 0.20 |
| 1-vessel disease, no(%) | 210 (52.9%) | 220 (53.9%) |  |  |
| 2-vessel disease, no(%) | 102 (25.7%) | 110 (27.0%) |  |  |
| LM or 3-vessel disease, no(%) | 82 (20.7%) | 76 (18.6%) |  |  |
| Other | 3 (0.8%) | 2 (0.5%) |  |  |
| **Hospital discharge treatment** |  |  |  |  |
| Aspirin, no(%) | 388 (97.7%) | 400 (97.8%) | 0.00 | -0.13, 0.14 |
| Calcium antagonist, no(%) | 73 (18.4%) | 73 (17.8%) | 0.01 | -0.12, 0.15 |
| Beta-blockers, no(%) | 45 (11.3%) | 388 (94.9%) | 3.1 | 2.9, 3.3 |
| ACEI, no(%) | 169 (42.6%) | 159 (38.9%) | 0.08 | -0.06, 0.21 |
| Statins, no(%) | 394 (99.2%) | 406 (99.3%) | 0.00 | -0.14, 0.14 |
| Diuretics, no(%) | 22 (5.5%) | 46 (11.2%) | 0.21 | 0.07, 0.35 |
| *^1^*Median (IQR); n (%) | | | | |
| *^2^*Standardized Mean Difference | | | | |
| *^3^*CI = Confidence Interval  PCI: percutaneous coronary intervention, CABG: coronary artery by-pass grafting, CPR: cardiopulmonary resuscitation, LM: left main, ACEI: Angiotensin-converting-enzyme inhibitors, ARB: Angiotensin receptor blockers. ITT: Intention-to-treat | | | | |

| **Table S2: Baseline EQ-5D, On-treatment** |  |  |  |  |  |
| --- | --- | --- | --- | --- | --- |
| **Variable** | **Overall**  N = 3373*^1^* | **No beta-blocker**  N = 1727*^1^* | **Beta-blocker**  N = 1843*^1^* | **Difference***^2^* | **95% CI***^2,3^* |
| **Demography** |  |  |  |  |  |
| Median age (IQR) - year | 64 (57, 71) | 64 (56, 71) | 64 (57, 71) | 0.00 | -0.07, 0.06 |
| Female sex, no(%) | 707 (21.0%) | 362 (21.5%) | 345 (20.4%) | -0.03 | -0.10, 0.04 |
| **Risk Factors** |  |  |  |  |  |
| Current smoker, no(%) | 712 (21.4%) | 376 (22.6%) | 336 (20.1%) | -0.06 | -0.13, 0.01 |
| Hypertension, no(%) | 1471 (43.6%) | 726 (43.2%) | 745 (44.0%) | 0.02 | -0.05, 0.08 |
| Diabetes, no(%) | 413 (12.3%) | 204 (12.2%) | 209 (12.4%) | 0.01 | -0.06, 0.07 |
| **Prior Cardiovascular Disease** |  |  |  |  |  |
| Previous AMI, no(%) | 190 (5.6%) | 101 (6.0%) | 89 (5.3%) | 0.03 | -0.04, 0.10 |
| Previous PCI, no(%) | 177 (5.3%) | 97 (5.8%) | 80 (4.7%) | 0.05 | -0.02, 0.11 |
| Previous CABG, no(%) | 38 (1.1%) | 16 (1.0%) | 22 (1.3%) | 0.03 | -0.03, 0.10 |
| Previous Stroke, no(%) | 67 (2.0%) | 35 (2.1%) | 32 (1.9%) | 0.01 | -0.05, 0.08 |
| Previous heart failure, no(%) | 18 (0.5%) | 13 (0.8%) | 5 (0.3%) | -0.07 | -0.13, 0.00 |
| **Presentation Characteristics** |  |  |  |  |  |
| Chest pain as main symptom, no(%) | 3266 (96.8%) | 1,624 (96.7%) | 1642 (97.0%) | 0.02 | -0.05, 0.09 |
| CPR before hospital, no(%) | 13 (0.4%) | 7 (0.4%) | 6 (0.4%) | 0.01 | -0.06, 0.08 |
| Heart rate, median (IQR), bpm | 74 (64, 84) | 73 (63, 84) | 75 (65, 85) | 0.06 | -0.01, 0.12 |
| Systolic blood pressure, median (IQR) | 152 (137, 170) | 152 (138, 169) | 152 (136, 170) | 0.00 | -0.06, 0.07 |
| Beta-blockers, no(%) | 304 (9.2%) | 144 (8.7%) | 160 (9.6%) | 0.03 | -0.04, 0.10 |
| **In-hospital Course** |  |  |  |  |  |
| Coronary angiography |  |  |  | 0.06 | -0.01, 0.13 |
| 1-vessel disease, no(%) | 1960 (58.2%) | 983 (58.6%) | 977 (57.8%) |  |  |
| 2-vessel disease, no(%) | 903 (26.8%) | 460 (27.4%) | 443 (26.2%) |  |  |
| LM or 3-vessel disease, no(%) | 475 (14.1%) | 220 (13.1%) | 255 (15.1%) |  |  |
| Other | 29 (0.9%) | 15 (0.9%) | 14 (0.8%) |  |  |
| **Hospital discharge treatment** |  |  |  |  |  |
| Aspirin, no(%) | 3,302 (97.9%) | 1648 (98.1%) | 1654 (97.7%) | 0.03 | -0.04, 0.10 |
| P2Y12 inhibitors, no(%) | 3289 (97.5%) | 1650 (98.2%) | 1639 (96.8%) | 0.09 | 0.02, 0.16 |
| Calcium antagonist, no(%) | 591 (17.5%) | 312 (18.6%) | 279 (16.5%) | 0.06 | -0.01, 0.12 |
| Beta-blockers, no(%) | 1722 (51.1%) | 83 (4.9%) | 1639 (96.8%) | 4.7 | 4.5, 4.8 |
| ACEI or ARB | 2707 (80.3%) | 1359 (80.9%) | 1348 (79.6%) | 0.03 | -0.04, 0.10 |
| Statins, no(%) | 3337 (99.0%) | 1655 (98.6%) | 1682 (99.4%) | 0.08 | 0.01, 0.14 |
| Diuretics, no(%) | 221 (6.6%) | 104 (6.2%) | 117 (6.9%) | 0.03 | -0.04, 0.09 |
| *^1^*Median (IQR); n (%) | | | | | |
| *^2^*Standardized Mean Difference  PCI: percutaneous coronary intervention, CABG: coronary artery by-pass grafting, CPR: cardiopulmonary resuscitation, LM: left main, ACEI: Angiotensin-converting-enzyme inhibitors, ARB: Angiotensin receptor blockers. PP: Per-protocol | | | | | |

| **Table S3: EQ-5D ITT, FU1** |  |  |  |  |
| --- | --- | --- | --- | --- |
| **Variable** | **Overall**  N = 4080*^1^* | **No beta-blocker**  N = 2057*^1^* | **Beta-blocker**  N = 2023*^1^* | **p-value***^2^* |
| EQ-5D INDEX, FU1 | 0.94 (0.88, 0.97) | 0.94 (0.88, 0.97) | 0.94 (0.88, 0.97) | >0.9 |
| Missing | 496 | 257 | 239 |  |
| MOBILITY |  |  |  | >0.9 |
| 1 | 3162 (87.3%) | 1592 (87.5%) | 1570 (87.1%) |  |
| 2 | 453 (12.5%) | 224 (12.3%) | 229 (12.7%) |  |
| 3 | 8 (0.2%) | 4 (0.2%) | 4 (0.2%) |  |
| Missing | 457 | 237 | 220 |  |
| SELF-CARE |  |  |  | 0.8 |
| 1 | 3554 (98.2%) | 1789 (98.4%) | 1765 (98.1%) |  |
| 2 | 61 (1.7%) | 28 (1.5%) | 33 (1.8%) |  |
| 3 | 4 (0.1%) | 2 (0.1%) | 2 (0.1%) |  |
| Missing | 461 | 238 | 223 |  |
| USUAL ACTIVITIES |  |  |  | 0.2 |
| 1 | 3232 (89.4%) | 1608 (88.5%) | 1624 (90.3%) |  |
| 2 | 351 (9.7%) | 191 (10.5%) | 160 (8.9%) |  |
| 3 | 32 (0.9%) | 18 (1.0%) | 14 (0.8%) |  |
| Missing | 465 | 240 | 225 |  |
| PAIN/DISCOMFORT |  |  |  | >0.9 |
| 1 | 2287 (63.4%) | 1146 (63.2%) | 1141 (63.7%) |  |
| 2 | 1203 (33.4%) | 608 (33.5%) | 595 (33.2%) |  |
| 3 | 115 (3.2%) | 59 (3.3%) | 56 (3.1%) |  |
| Missing | 475 | 244 | 231 |  |
| ANXIETY/DEPRESSION |  |  |  | 0.7 |
| 1 | 2426 (67.5%) | 1221 (67.6%) | 1205 (67.3%) |  |
| 2 | 1068 (29.7%) | 530 (29.4%) | 538 (30.1%) |  |
| 3 | 101 (2.8%) | 54 (3.0%) | 47 (2.6%) |  |
| Missing | 485 | 252 | 233 |  |
| *^1^*Median (IQR); n (%) | | | | |
| *^2^*Wilcoxon rank sum test; Fisher's exact test; Pearson's Chi-squared test  ITT = Intention-to-treat. FU= Follow-up | | | | |

| **Table S4: EQ-5D ITT, FU2** |  |  |  |  |
| --- | --- | --- | --- | --- |
| **Variable** | **Overall**  N = 4080*^1^* | **No beta-blocker**  N = 2057*^1^* | **Beta-blocker**  N = 2023*^1^* | **p-value***^2^* |
| EQ-5D INDEX, FU2 | 0.94 (0.88, 0.97) | 0.94 (0.88, 0.97) | 0.94 (0.88, 0.97) | 0.8 |
| Missing | 553 | 267 | 286 |  |
| MOBILITY |  |  |  | 0.4 |
| 1 | 3047 (85.7%) | 1557 (86.2%) | 1490 (85.2%) |  |
| 2 | 501 (14.1%) | 245 (13.6%) | 256 (14.6%) |  |
| 3 | 7 (0.2%) | 5 (0.3%) | 2 (0.1%) |  |
| Missing | 525 | 250 | 275 |  |
| SELF-CARE |  |  |  | 0.067 |
| 1 | 3501 (98.5%) | 1780 (98.5%) | 1721 (98.5%) |  |
| 2 | 49 (1.4%) | 22 (1.2%) | 27 (1.5%) |  |
| 3 | 5 (0.1%) | 5 (0.3%) | 0 (0.0%) |  |
| Missing | 525 | 250 | 275 |  |
| USUAL ACTIVITIES |  |  |  | 0.3 |
| 1 | 3240 (91.2%) | 1647 (91.2%) | 1593 (91.1%) |  |
| 2 | 277 (7.8%) | 136 (7.5%) | 141 (8.1%) |  |
| 3 | 37 (1.0%) | 23 (1.3%) | 14 (0.8%) |  |
| Missing | 526 | 251 | 275 |  |
| PAIN/DISCOMFORT |  |  |  | 0.6 |
| 1 | 2120 (59.7%) | 1068 (59.2%) | 1052 (60.2%) |  |
| 2 | 1284 (36.1%) | 665 (36.9%) | 619 (35.4%) |  |
| 3 | 148 (4.2%) | 71 (3.9%) | 77 (4.4%) |  |
| Missing | 528 | 253 | 275 |  |
| ANXIETY/DEPRESSION |  |  |  | 0.3 |
| 1 | 2625 (74.4%) | 1321 (73.8%) | 1304 (75.0%) |  |
| 2 | 824 (23.3%) | 422 (23.6%) | 402 (23.1%) |  |
| 3 | 81 (2.3%) | 48 (2.7%) | 33 (1.9%) |  |
| Missing | 550 | 266 | 284 |  |
| *^1^*Median (IQR); n (%) | | | | |
| *^2^*Wilcoxon rank sum test; Fisher's exact test; Pearson's Chi-squared test  ITT = Intention-to-treat. FU= Follow-up | | | | |

| **Table S5: EQ-5D, On-treatment, FU1** |  |  |  |  |
| --- | --- | --- | --- | --- |
| **Variable** | **Overall**  N = 3570*^1^* | **No beta-blocker**  N = 1727*^1^* | **Beta-blocker**  N = 1843*^1^* | **p-value***^2^* |
| EQ-5D INDEX, FU1 | 0.94 (0.88, 0.97) | 0.94 (0.88, 0.97) | 0.94 (0.88, 0.97) | 0.2 |
| Missing | 439 | 219 | 220 |  |
| MOBILITY |  |  |  | 0.5 |
| 1 | 2770 (87.4%) | 1345 (88.1%) | 1425 (86.8%) |  |
| 2 | 390 (12.3%) | 178 (11.7%) | 212 (12.9%) |  |
| 3 | 8 (0.3%) | 4 (0.3%) | 4 (0.2%) |  |
| Missing | 402 | 200 | 202 |  |
| SELF-CARE |  |  |  | 0.4 |
| 1 | 3108 (98.2%) | 1504 (98.5%) | 1604 (97.9%) |  |
| 2 | 53 (1.7%) | 21 (1.4%) | 32 (2.0%) |  |
| 3 | 4 (0.1%) | 2 (0.1%) | 2 (0.1%) |  |
| Missing | 405 | 200 | 205 |  |
| USUAL ACTIVITIES |  |  |  | 0.8 |
| 1 | 2840 (89.8%) | 1365 (89.5%) | 1475 (90.1%) |  |
| 2 | 295 (9.3%) | 147 (9.6%) | 148 (9.0%) |  |
| 3 | 27 (0.9%) | 13 (0.9%) | 14 (0.9%) |  |
| Missing | 408 | 202 | 206 |  |
| PAIN/DISCOMFORT |  |  |  | 0.5 |
| 1 | 2024 (64.2%) | 992 (65.2%) | 1032 (63.3%) |  |
| 2 | 1031 (32.7%) | 484 (31.8%) | 547 (33.6%) |  |
| 3 | 96 (3.0%) | 45 (3.0%) | 51 (3.1%) |  |
| Missing | 419 | 206 | 213 |  |
| ANXIETY/DEPRESSION |  |  |  | 0.9 |
| 1 | 2141 (68.2%) | 1038 (68.6%) | 1103 (67.8%) |  |
| 2 | 917 (29.2%) | 435 (28.8%) | 482 (29.6%) |  |
| 3 | 83 (2.6%) | 40 (2.6%) | 43 (2.6%) |  |
| Missing | 429 | 214 | 215 |  |
| *^1^*Median (IQR); n (%) | | | | |
| *^2^*Wilcoxon rank sum test; Fisher's exact test; Pearson's Chi-squared test  PP= Per-protocol. FU= Follow-up | | | | |

| **Table S6: EQ-5D, On-treatment, FU2** |  |  |  |  |
| --- | --- | --- | --- | --- |
| **Variable** | **Overall**  N = 3570*^1^* | **No beta-blocker**  N = 1727*^1^* | **Beta-blocker**  N = 1843*^1^* | **p-value***^2^* |
| EQ-5D INDEX, FU2 | 0.94 (0.88, 0.97) | 0.94 (0.90, 0.97) | 0.94 (0.88, 0.97) | >0.9 |
| Missing | 488 | 222 | 266 |  |
| MOBILITY |  |  |  | 0.2 |
| 1 | 2658 (85.7%) | 1309 (86.4%) | 1349 (84.9%) |  |
| 2 | 438 (14.1%) | 201 (13.3%) | 237 (14.9%) |  |
| 3 | 7 (0.2%) | 5 (0.3%) | 2 (0.1%) |  |
| Missing | 467 | 212 | 255 |  |
| SELF-CARE |  |  |  | 0.083 |
| 1 | 3055 (98.5%) | 1489 (98.3%) | 1566 (98.6%) |  |
| 2 | 42 (1.4%) | 20 (1.3%) | 22 (1.4%) |  |
| 3 | 5 (0.2%) | 5 (0.3%) | 0 (0.0%) |  |
| Missing | 468 | 213 | 255 |  |
| USUAL ACTIVITIES |  |  |  | 0.12 |
| 1 | 2832 (91.3%) | 1388 (91.7%) | 1444 (90.9%) |  |
| 2 | 240 (7.7%) | 107 (7.1%) | 133 (8.4%) |  |
| 3 | 30 (1.0%) | 19 (1.3%) | 11 (0.7%) |  |
| Missing | 468 | 213 | 255 |  |
| PAIN/DISCOMFORT |  |  |  | 0.3 |
| 1 | 1863 (60.1%) | 901 (59.6%) | 962 (60.6%) |  |
| 2 | 1107 (35.7%) | 555 (36.7%) | 552 (34.8%) |  |
| 3 | 130 (4.2%) | 57 (3.8%) | 73 (4.6%) |  |
| Missing | 470 | 214 | 256 |  |
| ANXIETY/DEPRESSION |  |  |  | 0.3 |
| 1 | 2300 (74.6%) | 1110 (73.8%) | 1190 (75.4%) |  |
| 2 | 711 (23.1%) | 354 (23.5%) | 357 (22.6%) |  |
| 3 | 72 (2.3%) | 41 (2.7%) | 31 (2.0%) |  |
| Missing | 487 | 222 | 265 |  |
| *^1^*Median (IQR); n (%) | | | | |
| *^2^*Wilcoxon rank sum test; Fisher's exact test; Pearson's Chi-squared test  PP= Per-protocol. FU= Follow-up | | | | |

| **Table S7. Ordinal regression model, ITT** |  |  |  |  |
| --- | --- | --- | --- | --- |
| **Model** | **N** | **OR***^1^* | **95% CI***^1^* | **p-value** |
| EQ5D time 1 | | | | |
| No beta blocker | 1,800 | — | — |  |
| Beta blocker | 1,784 | 1.00 | 0.89, 1.13 | >0.9 |
| EQ5D time 2 | | | | |
| No beta blocker | 1,790 | — | — |  |
| Beta blocker | 1,737 | 1.02 | 0.90, 1.15 | 0.8 |
| VAS time 1 | | | | |
| No beta blocker | 1,778 | — | — |  |
| Beta blocker | 1,764 | 1.06 | 0.94, 1.18 | 0.4 |
| VAS time 2 | | | | |
| No beta blocker | 1,757 | — | — |  |
| Beta blocker | 1,682 | 0.97 | 0.87, 1.09 | 0.7 |
| *^1^*OR = Odds Ratio, CI = Confidence Interval | | | | |

**Figure S1. Flowchart of inclusion to RQoL substudy**


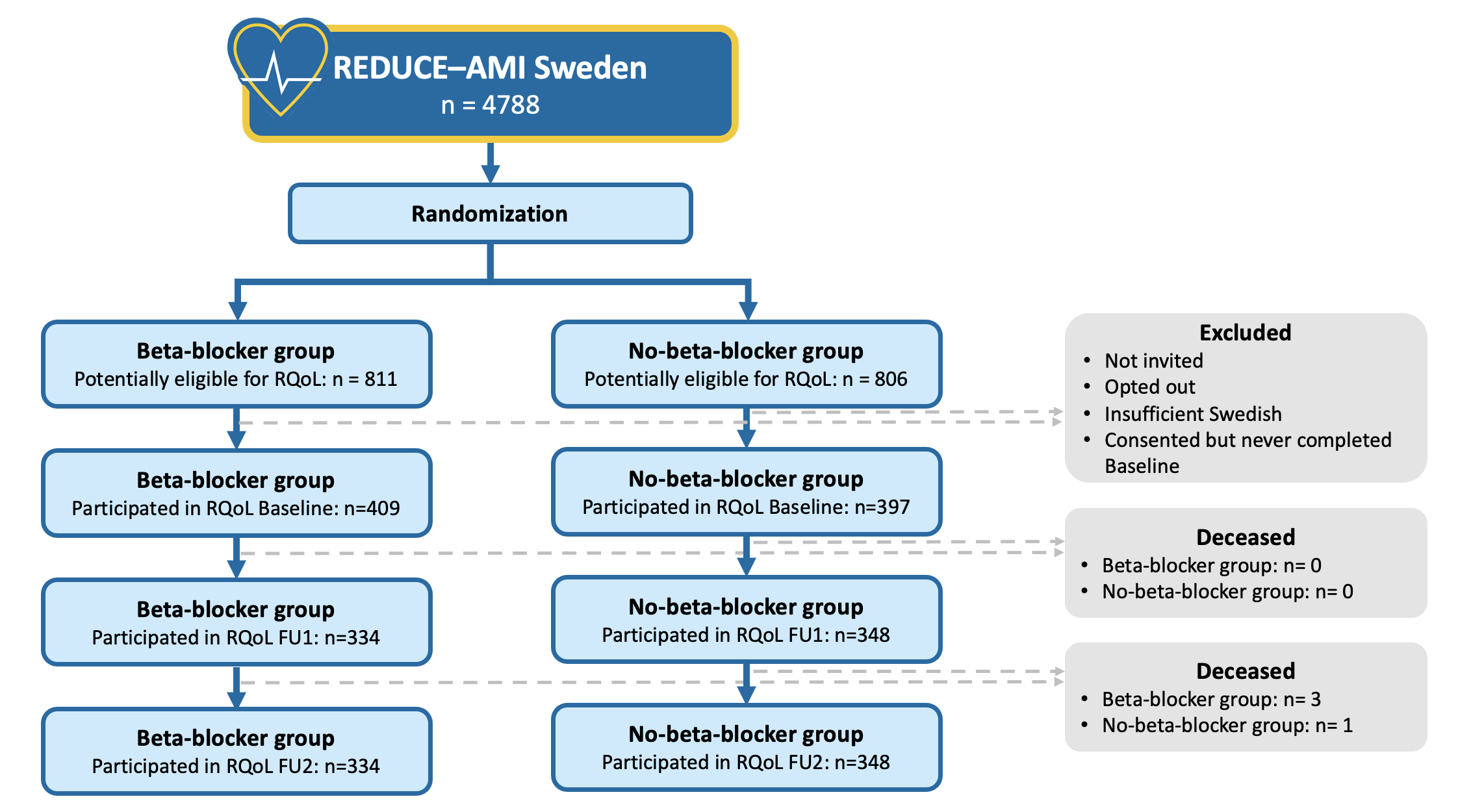


RQoL = REDUCE-Quality of Life. FU = Follow-up

**Figure S2. Subgroup analysis EQ-5D index score, 6-10 week follow-up**


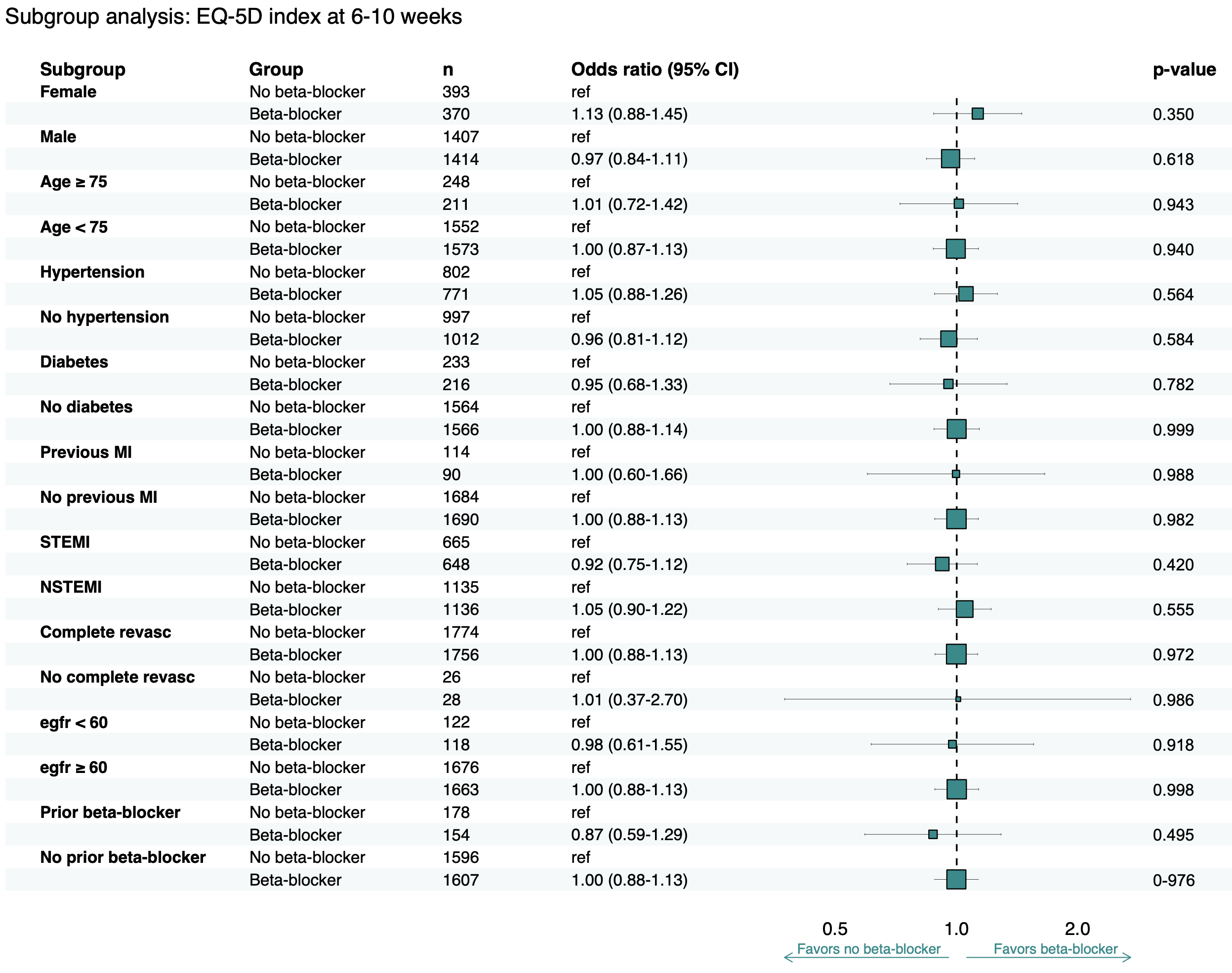


Subgroup analysis. EQ-5D index score stratified into subgroups according to intention-to-treat at 11-13 months after acute myocardial infarction.
